# 8p23.1 duplication syndrome differentiated from copy number variation of the defensin cluster at prenatal diagnosis in four new families

**DOI:** 10.1186/1755-8166-3-3

**Published:** 2010-02-18

**Authors:** John CK Barber, Dave Bunyan, Merryl Curtis, Denise Robinson, Susanne Morlot, Anette Dermitzel, Thomas Liehr, Claudia Alves, Joana Trindade, Ana I Paramos, Clare Cooper, Kevin Ocraft, Emma-Jane Taylor, Viv K Maloney

**Affiliations:** 1 Wessex Regional Genetics Laboratory, Salisbury NHS Foundation Trust, Salisbury, SP2 8BJ, UK; 2 National Genetics Reference Laboratory (Wessex), Salisbury NHS Foundation Trust, Salisbury, SP2 8BJ, UK; 3 Human Genetics Division, Southampton University School of Medicine, Southampton, SO16 6YD, UK; 4 Institute of Medical Genetics, University Hospital Wales, Cardiff, CF14 4XW, UK; 5 Medizinisches Versorgungszentrum wagnerstibbe, Georgstr 50, Hannover, Germany; 6 Institut für Humangenetik und Anthropologie, Jena University Hospital, Jena, Germany; 7 GDPN, Genetica Medica e Diagnostico Pre-natal, Porto, Portugal; 8 Prenatal Diagnosis Unit, District Hospital, Faro, Portugal; 9 Centre for Medical Genetics, Nottingham City Hospital, Nottingham, NG5 1PB, UK

## Abstract

**Background:**

The 8p23.1 duplication syndrome and copy number variation of the 8p23.1 defensin gene cluster are cytogenetically indistinguishable but distinct at the molecular level. To our knowledge, the 8p23.1 duplication syndrome has been described at prenatal diagnosis only once and we report our experience with four further apparent duplications ascertained at prenatal diagnosis.

**Methods:**

Additional material at band 8p23.1 was detected using conventional G-banded cytogenetics in each case. Multiplex Ligation-dependent Probe Amplification (MLPA) or Fluorescence In Situ Hybridisation (FISH) were used depending on whether only DNA (Cases 1 and 4) or cytogenetic preparations (Cases 2 and 3) were available from the laboratory of origin. The extent of the duplication in Case 1 was retrospectively determined using array Comparative Genomic Hybridisation (array CGH).

**Results:**

Three cases of 8p23.1 duplication syndrome were found (Cases 1 to 3). Two were *de novo *and continued to term and the third, a paternally transmitted duplication, was terminated because of a previous child with psychomotor delay and 8p23.1 duplication syndrome. Case 1 was ascertained with a hypoplastic left heart but the ventricular septal and interventricular defects, in Cases 2 and 3 respectively, were found after ascertainment for advanced maternal age. By contrast, case 4 was a maternally transmitted copy number variation of the defensin cluster with normal outcome.

**Conclusions:**

Our data underline the need to differentiate 8p23.1 duplications from copy number variation of the defensin cluster using FISH, MLPA or array CGH. Cardiac defects were ascertained by ultrasound in only one of the three duplication 8p23.1 pregnancies but were visible in two of the three at 21 to 22 weeks gestation. Our results provide further evidence that both deletion and duplication of the *GATA4 *transcription factor can give rise to a variety of conotruncal heart defects with variable penetrance and expressivity.

## Background

The application of array CGH is rapidly identifying new recurrent microdeletion and microduplication syndromes [1] and a previously unsuspected level of copy number variation which needs to be distinguished from pathogenic change [2]. Among these new syndromes is the 8p23.1 duplication between the 8p23.1 olfactory receptor/defensin repeats (ORDRs) at REPD in distal 8p23.1 (REPeat Distal) and REPP (REPeat Proximal) in proximal 8p23.1 (Table 1). This genomic disorder is the reciprocal of the 8p23.1 deletion syndrome [3] and has, to our knowledge, only been confirmed at the molecular level in four families to date [4,5]. Duplications of 8p23.1 have been associated with a variable phenotype that may include one or more of developmental delay, mild dysmorphism and heart defects. The single prenatal case had only mild dysmorphism and normal development at the age of 15 months with no evidence of a heart defect (Case 1 [5]).

**Table 1:** MLPA and BAC FISH results in Cases 1 to 4:

Band	BAC/MLPA*	Mb from telomere (hg 18 Build 36)	Case 1 Proband	Case 2 Proband	Case 3 Proband, sister and father	Case 4 Proband and mother
8p23.3	RP11-410N18	1,980,652-2,132,993	-	Normal	-	-

**8p23.2**	RP11-159F11	2,215,497-2,435,332	-	Normal	-	-

**8p23.2**	*CSMD1 *(4 probes)*	2,780,282-4,839,736	Normal	-	-	Normal

8p23.1	CTD-2629I16	6,684,740-6,685,317	-	Normal	Normal	-

8p23.1	ANGPT2*	6,347,22 -6,408,174	Normal	-	-	Normal

8p23.1	*DEFB1 *(2 probes)*	6,715,511-6,722,939	Normal	-	-	Normal

8p23.1	*DEFA6 *(2 probes)*	6,769,631-6,771,008	Normal	-	-	Normal

8p23.1	*DEFA4 *(2 probes)*	6,780,755-6,783,196	Normal	-	-	Normal

8p23.1	DEFA5*	6,900,239-6,901,669	Normal	-	-	Normal

**REPD**	RP11-594D21	7,105,087-7,258,467	-	Normal	-	-

**REPD**	RP11-122N11	7,295,548-7,305,838	-	**_**	**dup**	-

**REPD**	RP11-1118M6	7,286,844-7,462,059	-	**dup**	**_**	-

**REPD**	RP11-774P7	7,318,738-7,396,455	-	Normal	**_**	-

**REPD**	DEFB4 etc (10 probes)*	7,789,609-7,791,647 Complex	Normal	**_**	**_**	**trp**

8p23.1	RP11-211C9	8,504,285-8,677,721	**_**	**dup**	**dup**	-

8p23.1	MFHAS1 (MASL1)*	8,679,409-8,788,541	**dup**	**_**	**_**	Normal

8p23.1	PPP1R3B*	9,031,186-9,045,630	**dup**	**_**	**_**	Normal

8p23.1	TNKS*	9,450,855-9,677,266	**dup**	**_**	**_**	Normal

8p23.1	MSRA*	9,949,189-10,323,803	**dup**	**_**	**_**	Normal

8p23.1	BLK*	11,388,930-11,459,516	**dup**	**_**	**_**	Normal

8p23.1	GATA4*	11,599,162-11,654,918	**dup**	**_**	**_**	Normal

8p23.1	RP11-589N15	11,627,380-11,803,128	-	**dup**	**dup**	-

**REPP**	RP11-351I21	12,233,365-12,434,472	-	**dup**	**_**	-

**REPP**	RP11-24D9	12,433,487-12,590,982	-	-	**dup**	-

**8p22**	RP11-433L7	14,278,096-14,461,154	-	-	Normal	-

**8p22**	MSR1*	16,009,758-16,094,671	Normal	-	-	Normal

8p21.3	CGAT1*	19,305,952-19,584,374	Normal	-	-	Normal

Letters in bold highlight the G-dark bands, REPD and REPP and the results with a change in copy number.

Dashes indicate probes "not tested" and longer dashes (in bold) those "not tested" but expected to show copy number change based on the other results.

BAC names are given without italics and the genes targeted by MLPA probes in italics.

The REPD and REPP repeats mediate a remarkable variety of simple and complex chromosome rearrangements [6] and are themselves copy number variable with 2 to 7 copies of the beta defensin components in the normal population [7]. Numbers as high as 9 to 12 become cytogenetically visible as "euchromatic variants" [7] that are only associated with a predisposition to psoriasis [8]. These high level copy number variations are cytogenetically indistinguishable from the 8p23.1 duplications [4] and both the copy number variants and genuine duplications can be transmitted from parents to children. Here we report on our experience of using follow-up MLPA and FISH testing of apparent cytogenetic duplications of 8p23.1 detected during prenatal diagnosis. The four cases include two *de novo *duplications, a paternally transmitted duplication of 8p23.1 and a benign maternally transmitted defensin copy number variation.

## Methods

Amniotic fluid cells were cultured, G-banded and analysed using established techniques. Quantitative Fluorescent Polymerase Chain Reaction (QF-PCR) analysis was performed using an autosomal multiplex according to a method adapted from Mann et al [9] (Case 1). DNA was extracted using a Qiagen EZ1 machine and MLPA [10] was carried out according to the manufacturer's instructions with the P139 defensin kit which contains 29 probes mapping across the distal short arm of chromosome 8 (Cases 1 and 4) (Table 1) (please see the MRC-Holland web site for further information). MLPA PCR products were separated on an ABI 3100 Sequencer, analysed using Applied Biosystems Inc Genotyper version 2.0 (Table 1) and the results collated in an in-house Excel spreadsheet as previously described [11]. Array CGH was carried out with the BlueGnome Cytochip Focus BAC array and BlueFuse software according to the manufacturer's instructions with minor modifications [12] (Case 1). FISH was carried out using standard methods with Ensembl 37 k cloneset bacterial artificial chromosomes (BACs) (Table 1) chosen from the Ensembl web browser http://www.ensembl.org/Homo_sapiens/Location/Genome (Cases 2 and 3). The BACs were grown, validated and prepared for FISH by the National Genetics Reference Laboratory (Wessex). Additional BAC FISH was performed in Case 2 as previously reported [13].

## Results

### Case and family reports

Case 1 (*de novo *pathogenic 8p23.1 duplication): A G2P1 lady of 31 was referred for amniocentesis at 21+2 weeks gestation after a hypoplastic left heart (HLH) has been detected with ultrasound in her unborn daughter. Her previous son (with a different partner) had been phenotypically normal at term and there was no family history of congenital heart defects. She was a non-smoker who had taken no alcohol or drugs during pregnancy. Following genetic counselling at 25 weeks, the parents decided to continue the pregnancy and an infant girl was delivered at 41 weeks gestation with apgar scores of 7 at 1 min and 9 at 5 min. This girl weighed 3.3 kg (50th centile), was 53 cm long (just above 50th centile) and had a head circumference of 38 cm (97th centile). She underwent successful first stage Norwood surgery for HLH at the age of 2 days. On examination, at just under 3 months of age, she was only 4.26 kg in weight (0.4th centile) and 55 cm in length despite tolerating her feeds well. She was being treated with Cephalexin for an E. Coli infection but was otherwise considered well. A transthoracic echocardiogram showed a corrected atrial septum, mild right atrioventricular valvular regurgitation, no neo-aortic regurgitation, an RV-PA conduit maximum velocity (V max) of 3.7 m/sec, arch turbulence V max of 2.7 m/sec and good ventricular function. Blood pressure was 60/50 in the right leg with saturations of 82% in air. Cardiovascular examination revealed a single second heart sound and normal first heart sounds. There was a 3/6 ejection systolic murmur in the left upper sternal area. The chest was clear and her abdomen soft. Cardiac catheterization was planned. The mother reported no breathlessness in her daughter and had no other concerns.

Case 2 (*de novo *pathogeneic 8p23.1 duplication): A lady of 38 was referred for prenatal diagnosis at 18 weeks gestation because of her advanced maternal age. No anomalies were seen with ultrasound at the time but a muscular VSD was detected during an ultrasound scan at 22 weeks. A boy was delivered at 41 weeks and one day of pregnancy with weight 2920 g (10-25th centile), length 48 cm (10-25th centile) and OFC 35 cm (50th centile). Apgar scores were 10/10/10. He was healthy and had no dysmorphic stigmata. Sonographic investigation of the brain was normal. Cardiac echogram showed a muscular VSD, a small bidirectional shunt, PDA, an open foramen ovale, thickened aortic valve and no stenosis. At two months of age a systolic heart murmur was noted.

Case 3 (pathogenic paternally inherited 8p23.1 duplication): A G4P2A1 lady was referred for prenatal diagnosis, during her fourth pregnancy, due to an advanced maternal age of 35. No ultrasound anomalies were recorded. The mother was a healthy Caucasian who had been through secondary education. Her family history included a mentally retarded brother, who had died at the age of 35 (of unknown causes), and a maternal nephew with learning difficulties who had only attended primary school. Her karyotype was normal.

The mother's first pregnancy had ended in a spontaneous abortion at 20 weeks gestation. No fetal pathology records were available. During her second pregnancy, the mother had been hospitalized at 25 weeks gestation because of the threat of an early delivery. The pregnancy resulted in the eutocic pre-term delivery of a female of 2780 g with an apgar score of 9 at 1 minute. This girl was hospitalized for two days after birth with a systolic II/IV heart murmur, hyperbilirubinemia, clinical sepsis and a benign congenital cardiopathy consisting of a bicuspid aortic valve and very slight valvular pulmonary stenosis. Global developmental delay was diagnosed when she was 8 years old and she had special educational needs. Her 8p23.1 duplication was identified after the same duplication was found in her mother's fourth pregnancy (see below) and, at 15 years of age, she presented with global developmental delay, psycho-motor delay, speech impairment (dysarthrophonia) and cognitive and socio-emotional difficulties (selective mutism). The mother's 3rd pregnancy ended in a dystocic full term delivery of a healthy girl of 3200 g. This child had normal development and a normal karyotype.

The family was studied after the 8p23.1 duplication was found in the mother's fourth pregnancy and 8p23.1 duplications were identified in the father and this couple's first liveborn child. The pregnancy was legally terminated at 24 weeks due to the paternally inherited 8p23.1 duplication identified in the fetus and the psychomotor development delay found in their first child with the same duplication. Macroscopic fetal pathology of the male fetus revealed a left hydroureter and hydronephrosis of the kidney, a meso-septal interventricular defect of the heart and cerebral oedema of the brain. Microscopically, nodular hyperplasia of the adrenal cortex, pleural oedema, bilateral dilatation of the alveoli with disruption of the alveolar walls and an emphysema-like presentation were observed. The weight and maturity of the placenta were equivalent to a later gestational age of 29 weeks with evidence of oedema and villitis of unknown etiology.

The father was a Caucasian of 45 years of age with no relevant family history. He had bilateral conductive hearing loss and exostoses which had been surgically removed without clinical improvement. He had only attended primary education and the referring physician described him as "slow". He had the same duplication of 8p23.1 that had been transmitted to his daughter.

Case 4 (benign maternally inherited defensin copy number variant): A 27 year old lady was referred at 16+6 weeks gestation for prenatal diagnosis because of an increased risk of Down syndrome estimated, by nuchal translucency determination, at a combined risk of 1 in 235. The pregnancy continued and a phenotypically normal girl was born at term.

### Molecular cytogenetic and molecular genetic results

#### Case 1

**Figure 1:**
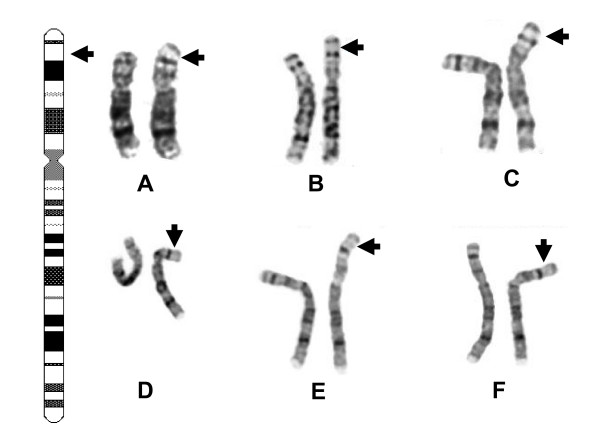
**G-banded partial karyotypes (A-F)**. (A) Case 1, (B) Case 2, (C) the Case 3 proband, (D) the father of the proband, (E) the elder sister of the proband and (F) Case 4. The duplicated or variant chromosome is on the right hand side of each chromosome pair and the expanded G-light region of 8p23.1 indicated by the black arrow in each case. Note the similarity of the G-banded copy number variant 8 in Case 4 to the duplicated 8 s in the probands of Cases 1 to 3.

**Figure 2:**
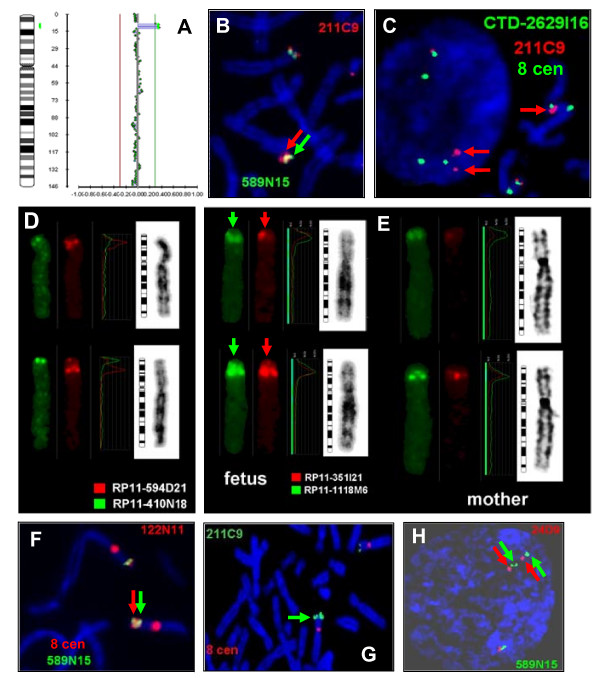
**Molecular cytogenetic results in Cases 1 to 3**. Case 1 (A): The BAC array CGH result, which confirmed the MLPA findings, displayed with BlueFuse software and showing the region of copy number gain at 8p23.1 (green bar to the right of the idiogram); Case 2 (B-E): (B) the larger metaphase signals (arrowed) from the 8p23.1 BACs RP11-211C9 (red) and RP11-589N15 (green) and (C) the enhanced signal strength from BAC RP11-211C9 (red) at metaphase (single red arrow) and the duplicated signals at interphase (double red arrows) (note, the green signals are from BAC CTD-2629I168 and the 8 centromere which both had normal copy number); (D) the normal results from BAC RP11-594D21 (red) in distal REPD and a control BAC RP11-410N18 from 8p23.3 (green); (E, left hand pairs) the duplicated signals (vertical arrows) from BACs RP11-351I21 (red) in REPP and RP11-1118M6 (green) in REPD from the fetus and (E, right hand pairs) the normal copy in the mother. Case 3 (F-H): (F) the enhanced metaphase signal strength (red and green arrows) from the REPD BACs RP11-122N11 (red) and RP11-589N15 (green) note, (the additional red signals are from the 8 centromere); (G) the duplicated metaphase signals (single green arrow) from BAC RP11-211C9 (green) and (H) at prometaphase (red and green arrows) from BAC RP11-24D9 (red) in REPP from BAC RP11-589N15 (green).

QF-PCR analysis showed no evidence of trisomy 13, 18 or 21 and FISH investigation of cultured cells with the Vysis *TUPLE1 (HIRA) *probe for 22q11.2 showed a normal hybridisation pattern. However, conventional cytogenetic analysis showed a duplication within the short arm of chromosome 8 at 8p23.1 (Figure 1A). The abnormality was confirmed in a fetal blood sample. Parental blood karyotypes were normal and the duplication had arisen *de novo*. MLPA analysis showed that 6 of the 29 probes located between REPD and REPP, including *GATA4*, were duplicated. Retrospective BAC array CGH analysis revealed increased average intensity ratios for a 3.87- 6.12 Mb region spanning 6 clones from RP11-347L3 to RP11-247B12 (Figure 2A). In accordance with ISCN 2009 [14], the karyotype was: 46, XX, dup(8)(p23.1p23.1)dn.mlpa 8p23.1(P139)x3.arr 8p23.1(RP11-347L3-RP11-247B12)x3.

#### Case 2

A duplication of 8p23.1 was suspected during conventional chromosome analysis of the amniotic fluid cultures (Figure 1B) and confirmed using FISH with a total of nine BACs. Only BACs mapping to and between REPD and REPP were duplicated (Figure 2B-2E) (Table 1). BAC RP11-594D21 from distal REPD gave a normal result (Figure 2D) while REPD BAC RP11-1118M6 was duplicated (Figure 2E) and RP11-774P7 gave a normal result despite being proximal to RP11-1118M6 (data not shown). This may reflect additional structural complexity or uncertainties in the assembly of the human genome in this sequence gap. Normal karyotypes were found in both parents and the duplication was de novo. The karyotype of the fetus was: 46, XY, dup(8)(p23.1p23.1)dn.ish dup(8)(RP11-410N18+,RP11-159F11+,CTD-2629I16+, RP11-594D21+, RP11-1118M6++, RP11-774P7+,RP11-211C9++,RP11-589N15++, RP11-351I21++).

#### Case 3

A duplication of 8p23.1 was suspected during conventional chromosome analysis of the amniotic fluid cultures (Figure 1C) and confirmed using FISH with six BACs. Both the BACs which map to either end of the interval between REPP and REPD were duplicated (Figure 2F-2H) as were the BACs which map to the REPP and REPD repeats (Figure 2F and 2H) (Table 1). Conventional chromosome analysis on the father (Figure 1D) and the family's eldest daughter (Figure 1E) showed the same duplication which was confirmed using FISH on peripheral blood from the father and daughter using the same set of BACs (Table 1). The mother had a normal karyotype and the normal karyotype of her middle daughter was confirmed using FISH. The karyotype of this boy was: 46, XY, dup(8)(p23.1p23.1)pat.ish dup(8)(CTD-2629I16+,RP11-122N11++,RP11-211C9++,RP11-589N15++,RP11-24D9++,RP11-433L7+).

#### Case 4

A duplication of 8p23.1 was suspected during conventional chromosome analysis of the amniotic fluid cultures (Figure 1F) and a similar chromosomal pattern was seen in the mother. The normal results at the six genes between REPD and REPP specifically excluded a duplication of 8p23.1 using MLPA with DNA from the fetus and mother. However, there was clear evidence for at least four copies (triplication) of the 8 genes within the copy number variable defensin cluster in both the fetus and mother (data not shown). These include *DEFB4*, *SPAG11*, *DEFB103A*, *DEFB104*, *DEFB105*, *DEFB106*, *DEFB107 *and *DEFB108*. This copy number variation had been transmitted from the phenotypically normal mother and the pregnancy continued. In accordance with ISCN 2009 [14], the karyotype of the fetus was: 46, XX, var(8)(p23.1p23.1).mlpa 8p23.1(P139)x4 mat.

## Discussion

**Figure 3:**
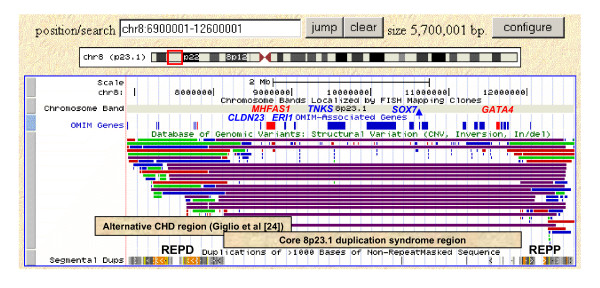
**Annotated screenshot of 5.7 Mb of band 8p23.1 (UCSC Genome Browser on Human Mar. 2006 Assembly (hg18))**. From bottom to top: the Segmental Duplications that contain the Olfactory Receptor and Defensin Repeats (ORDRs) are labelled REPD and REPP; the ~3.75 Mb core 8p23.1 duplication syndrome interval between REPD and REPP [5] and the ~2.5 Mb alternative CHD region proposed by Giglio et al [24] are illustrated by annotated boxes; the multiple copy number variations of REPD and REPP in the Database of Genome Variants (DGV) http://projects.tcag.ca/variation/ is indicated by the red, blue and green lines and the common polymorphic inversion between REPD and REPP by the purple lines; OMIM Morbid genes appear as red boxes and other OMIM genes as blue boxes (or lines); acronyms for the OMIM genes specifically mentioned in the text have been added above in corresponding colours. Note that the DGV does not contain any CNVs that match the 8p23.1 duplication syndrome region.

We have presented four prenatal cases in which an 8p23.1 duplication was suspected on cytogenetic grounds. MLPA or FISH confirmed 8p23.1 duplication syndrome in Cases 1 to 3 and only copy number variation of the defensin cluster in Case 4. Cases 1 and 2 were *de novo*, the duplication in Case 3 was directly transmitted from the father and the copy number variation in Case 4 was maternally transmitted. It is reasonable to conclude that Cases 1 to 3 all had a core duplication of ~3.75 Mb between the proximal and distal ORDRs (REPD and REPP) as shown using array CGH in Case 1 (Figure 2A, Figure 3). When these cases are added to those in the literature, the 8p23.1 duplication has now been confirmed, using molecular cytogenetic methods, in eleven individuals of whom four cases were de novo and another four had duplications transmitted from a father and two mothers (Table 2). An estimate of the prevalence of this condition can be derived from a recent series of 2,419 diagnostic patients analysed using oligonucleotide array CGH [15]; one dup(8)(p23.1p23.1) was found compared with sixteen 22q11.2 DiGeorge/Velocardiofacial syndrome (DG/VCFS) deletions. As DG/VCFS has a population frequency of ~1 in 4,000 [16], the 8p23.1 duplication syndrome has an estimated prevalence of 1 in 64,000. No examples of the full 8p23.1 duplication syndrome region have been reported among the 29,133 CNVs currently in the Database of Genome Variants (DGV) http://projects.tcag.ca/variation/ (Figure 3).

**Table 2:** Features of the present and previous cases of the 8p23.1 duplication syndrome:

Physical findings at birth or diagnosis		Present Case 1	Present Case 2	Present Case 3 Proband	Present Case 3 Sister	Present Case 3 Father	Barber *et al*. (2008) Case 1	Barber *et al*. (2005) Case 1	Barber *et al*. (2008) Family 1 Proband	Barber *et al*. (2008) Family 1 Mother	Barber *et al*. (2008) Family 2 Proband	Barber *et al*. (2008) Family 2 Mother
Ascertainment of dup(8)		CHD	AMA	AMA	AMA	AMA	1:150 risk	DD;CHD	PNAI	Daughter	DYS	Son

Prenatal/ Postnatal		Pre	Pre	Pre	Pre	Pre	Pre	Post	Post	Post	Post	Post

Pregnancy continued		Y	Y	N	n/a	n/a	Y	n/a	n/a	n/a	n/a	n/a

Sex		F	M	M	F	M	F	F	F	F	M	F

Delivery gestation (wks)		41	41+1	22	< 40	n/a	40	n/k	42	n/a	40+5	n/a

Apgar scores		7;9	10;10;10	n/a	9	n/a	8;9	n/a	n/a	n/a	n/a	n/a

Birth weight (kg)		3.3	2.92	n/r	2.78	n/k	3.15	n/k	3.6	?	3.39	?

OFC (cm)		38	35	n/r	?	n/k	33.6	n/k	n/r	n/k	39.5	?

Age at examination		3/12	Neonate	22/52	15	45	15/12	8	4	n/r	22/12	n/r

Developmental delay		n/a	n/a	n/a	++	?	n	+	-	-	-	+

Learning difficulties		n/a	n/a	n/a	+	+	n/a	+	-	+	-	+

Facial dysmorphism		-	-	-	-	-	+	+	+/-	+	++	++

Congenital heart defects		++	++	+	++	-	n	+	+	-	-	-

Neurological defects		-	n	?+	+	-	n	-	+	-	-	-

Syndactyly		-	-	-	-	-	-	-	-	-	+	+

Adrenal anomalies		-	-	+	-	-	-	-	++	-	-	-

Hydronephrosis and hydroureter		-	-	+	-	-	-	-	-	-	-	-

Alveolar anomalies		-	-	+	-	-	-	-	-	-	-	-

Hearing loss		-	-	-	-	+	-	-	-	-	-	-

Exostoses		-	-	-	-	+	-	-	-	-	-	-

AMA: Advanced Maternal Age; CHD: Congenital heart defect; DD: Developmental delay;

DYS: Facial dysmorphism; n: no evidence; n/a: not applicable; n/k: not known; n/r: not recorded; PNAI: Primary Neonatal Adrenal Insufficiency; Y = Yes; + = present; - = absent.

A summary of the phenotypic data on the eleven patients with 8p23.1 duplication syndrome is provided in Table 2. Ascertainment has been as a result of congenital heart disease (CHD) in only one of four prenatal cases and one of three postnatal probands but is now the most common single feature having been found in 6/11 individuals. Developmental delay and/or learning difficulties have been found in 5/11 but, of the remaining 6/11, one prenatal case was developmentally normal at 15 months of age and the three prenatal cases reported here have not yet reached an age at which this can assessed. A variable degree of facial dysmorphism was also present in 5/11 individuals. These results are broadly in line with those of Tsai et al [17] but, unfortunately, their results rely on cytogenetics alone and do not differentiate between the duplications and copy number variants. By contrast, partial toe syndactyly has been found in only one mother and son and adrenal anomalies in two probands but not in the mother, of one of these two, who had the same duplication.

Excluding the copy number variable regions, REPP and REPD, the duplicated interval contains 57 genes of which 34 are known and 23 are novel. These include the two transcription factors *GATA4 *and *SOX7 *and three micro-RNA loci. Deletions and heterozygous loss of function mutations of the GATA binding protein 4 gene (*GATA4*, OMIM *600576) are already strongly associated with conotruncal and septal heart defects [3,18-23] and it has been proposed that duplication of *GATA4 *is responsible for the pulmonary atresia and Tetralogy of Fallot found in two of the four published probands with 8p23.1 duplication syndrome [4,5]. The idea that *GATA4 *is responsible for the heart defect component of the 8p23.1 duplication syndrome is strengthened by the hypoplastic left heart in Case 1, the complex VSD in Case 2, the meso-septal interventricular heart defect in Case 3, at autopsy, and the mild heart defect in the eldest sister of Case 3.

The existence of a second heart disease gene in a 5-cM region of 8p23.1 between WI-8327 and D8S1825 (6,469,539 to 8,962,119 base pairs according to UCSC, March 2006) was proposed by Giglio et al [24] (Figure 3). However, the overlap between this ~2.5 Mb region and the REPD to REPP interval contains only four single copy genes (Figure 3), of which neither *PRAGMIN*, *CLDN23 *(*OMIM 609203), *MFHAS1 *(OMIM *605352) nor *ERI1 *(OMIM *608739) are currently good candidates for heart disease. Thus, it seems more likely that the absence of heart disease in some 8p23.1 duplication syndrome probands [5], four members of a family with a 133 kb microduplication of the *GATA4 *gene [25] and seven individuals with a 4.37 Mb duplication of 8p23.1 to 8p22 that included *GATA4 *[26], is more likely to reflect non-penetrance rather than the existence of a further heart disease gene between REPP and REPD. Normal heart development is thought to require interaction between *GATA4 *and the T-Box 5 (*TBX5*) gene [19,27] which suggests that variation in *TBX5*, or other genes involved in the development of the heart, might modify the consequences of altered *GATA4 *dosage. We conclude that both duplication and deletion of the *GATA4 *gene can give rise to a variety of conotruncal and septal heart defects but with variable penetrance and expressivity.

Other candidate genes derived from atypical microdeletions of proximal 8p23.1 may be considered candidate genes for features of the 8p23.1 duplication syndrome [3,28] (Figure 3). These include the TRF1-interacting, ankyrin related ADP-ribose polymerase gene (*TNKS*, OMIM*603303) for behavioural difficulties, and the SRY-Box 7 transcription factor (*SOX7*, OMIM *612202) for the developmental delay, as mutations of the related *SOX3 *gene have been associated with X-linked mental retardation [28]. By contrast, the diaphragmatic hernia found in a number of patients with the reciprocal deletion syndrome [29-31] has not, to our knowledge, been recorded in any 8p23.1 duplication syndrome patient to date.

There was evidence, using FISH, for both REPD and REPP being included in the duplication in Case 2 (Figure 2E) and Case 3 (Figure 2F and 2H) but not for the inclusion of either repeat in Case 1 using MLPA. REPP was also implicated in a previously published case (Family 1 [5]). Altered copy number might be expected at either or both repeats if the reciprocal deletions and duplications are generated by ectopic recombination (or NAHR) between the repeats [6]. Alternatively, altered copy number may be due to independent copy number variation of the ORDRs themselves.

Benign copy number variation of the defensin cluster was found in over 14% of a recent series of 1,275 patients analysed using array CGH [32], but cytogenetically visible amplifications, of the kind found in Case 4, are uncommon. These segregate in families with no significant clinical or reproductive effects other than a predisposition to Crohn's disease at low copy number [33] and psoriasis at high copy number [8]. Most chromosomes 8 have two copies of the defensin cluster and most individuals a total of four [34]. Thus, the triplication of the defensin cluster, relative to control DNA, implies a total of 12 copies in the Case 4 fetus and her mother. If the normal chromosome 8 had 2 copies, the variant chromosome would have 10 copies of the ORDR repeat and, as the repeat is a minimum of 240 kb in size [7], the ORDR array would extend to at least 2.4 Mb and thus become cytogenetically visible in the light microscope (Figure 1F) [4,7].

Both FISH and MLPA have been reliably used to confirm or exclude an 8p23.1 duplication between REPD and REPP. However, even normal chromosomes 8 can look duplicated with BACs that map to these repeats and thus differential signal strength between FISH probes does not constitute proof that copy number variation of REPD or REPP is the cause of an increase in the size of the 8p23.1 band. Recent evidence also suggests that the defensin clusters are switched between REPD to REPP by the polymorphic inversion between them [35], and this may be expected to change the appearance of the FISH signals seen on homologous pairs of chromosome 8 even if their copy number is the same or similar. Array CGH will also discriminate the duplication from the variant, and exclude additional imbalances, but careful choice of control samples may be required to accurately confirm the extent of the defensin copy number variation in all cases.

## Conclusions

In conclusion, our results underline the need to distinguish the 8p23.1 duplication from benign defensin copy number variation at prenatal diagnosis. Direct transmission of duplications and copy number variants from a parent to a child has been found on multiple occasions and transmission does not therefore discriminate between copy number variations of the defensin cluster and the 8p23.1 duplication syndrome. Cardiac defects were ascertained by ultrasound in only one of the three duplication 8p23.1 pregnancies but were visible in two of the three at 21 to 22 weeks gestation. Phenotypic data also indicate a relatively mild but variable syndrome and support the idea that duplication of the *GATA4 *transcription factor can give rise to a variety of conotruncal or septal heart defects with variable penetrance and expressivity.

## List of abbreviations

AMA: Advanced maternal age; BAC: Bacterial Artificial Chromosome; array CGH: array Comparative Genomic Hybridisation; CHD: Congenital heart disease; DGV: Database of Genome Variants; DNA: De-oxyo Ribose Nucleic Acid; FISH: Fluorescence In Situ Hybridisation HLH: Hypoplastic Left Heart; MCA: Multiple Congenital Anomalies; Multiple Ligation-dependent Probe Amplification (MLPA); OFC: Occipito-Frontal Circumference; PDA: Patent Ductus Arteriosus; ORDR: Olfactory Receptor and Defensin Repeat; Patent Ductus Arteriosus; PNAI: Primary Neonatal Adrenal Insufficiency; QF-PCR: Quantitative Fluorescent Polymerase Chain Reaction; REPD: REPeat Distal; REPP: REPeat Proximal; VSD: Ventricular Septal Defect.

## Competing interests

The authors declare that they have no competing interests.

## Authors' contributions

JCKB assembled the results and drafted the manuscript; DB carried out the MLPA analyses on Cases 1 and 4; MC and DR provided the laboratory and clinical information on Case 1; SM and AD provided the laboratory and clinical information on Case 2 and TL contributed the additional FISH results; CA, JT and AIP provided the laboratory and clinical information on Case 3 and her family; CC and KO provided the laboratory and clinical information on Case 4; EJT drafted Table 2; VKM prepared and validated the FISH probes and carried out the FISH analyses on Cases 2 and 3. All authors read and approved the final manuscript.
